# Comparative radioprotective effects of ascorbic acid and telmisartan, alone and in combination, on gamma-irradiated mandibular bone: an experimental rat study

**DOI:** 10.1007/s44445-026-00148-w

**Published:** 2026-04-25

**Authors:** Lobna M. Abdel-Aziz, Radwa M. Esmail, Heba Abdelfatah, Amira I. Sayed

**Affiliations:** 1 Faculty of Dental Medicine for Girls, Al-Azhar University, Nasr city, Cairo, Egypt; 2 Misr University for Science and Technology, 6th October, Giza, Egypt; 3 National Centre for Radiation Research and Technology, Egyptian Atomic Energy Authority, Cairo, Egypt; 4 Faculty of Oral and Dental Medicine, Nile Vally University, Fayoum, Egypt

**Keywords:** Gamma radiation, Radioprotectors, Ascorbic acid, Telmisartan, CBCT

## Abstract

This study was purposed to assess the radioprotective influences of ascorbic acid and Telmisartan, solitary and in combination, on rats exposed to gamma radiation. After different treatment regimens, the study assessed radiographic and histopathological outcomes in bone tissue. Four groups of forty male albino rats were created casually (*n* = 10). All groups were subjected to fractionated gamma radiation (6 Gy), 2 Gy day after day, three times per week. Group 1 (R) was subjected to fractionated gamma radiation only. Group 2 (MR) received Telmisartan 12 mg/kg orally once/day for 7 days before radiation. Group 3 (CR) received 200 mg/kg orally once/day for 7 days, Ascorbic Acid before radiation. Group 4 (MCR) received Telmisartan 12 mg/kg orally once/day for 7 days, and Ascorbic Acid 200 mg/kg orally once/day for 7 days before radiation. All groups were divided into two subgroups based on two-time intervals (3 days and 10 days). The efficacy of the treatments was evaluated through histological analysis and CBCT, or cone beam computed tomography, for bone density examination. Radiographically, the highest values of bone density measured after 3 days were for the MCR group**,** while the lowest values of bone density measured after 3 days were for the R group. The histological examination revealed a notable distinction between the 4 groups, with enhanced early matrix deposition in the MCR group at 3 days. The Haversian canals appeared wide and filled with RBCs; also, many reversal lines can be easily noticed and showing that the bone is highly compact with stenosis of Haversian canals at 10 days. A Combination of ascorbic acid with telmisartan demonstrated superiority over ascorbic acid and telmisartan monotherapy.

## Introduction

Radiation therapy, either by itself or combined with any other treatments as chemotherapy and/or surgery, has demonstrated its effectiveness in treating head and neck cancers, with a current high cure rate of 80% (Chen and Kuo 2017). In spite of their great efficacy in treating cancer, they have several negative adverse consequences that could lower the patient's standard of living and dental health (Hall et al. 2016).

There are two types of these detrimental effects: early and late. Early adverse effects can happen during the course of treatment or right after it is finished, while late adverse reaction occurs after months to a year of radiation treatment and may be permanent (Hall et al. 2016).

The radiobiological effect of radiation (IR) on the tissues occurs with both direct and indirect mechanisms. The direct mechanism of radiotherapy directly affects the DNA molecule, causing either simple base damage or a DNA double-strand break that may not be fully repaired or mis-repaired, leading to cell death. While the indirect effect decomposes water molecules, causing the release of free radicals, which may cause harm to the cell nucleus (Karbownik and Reiter 2000).

These free radicals destroy tissue through a variety of processes, such as tumor necrosis factor-α and transforming growth factor-β, which cause tissue damage and persistent inflammation. (Malathi et al. 2011, and Yağan et al. 2014). It was stated that two-thirds of the biological damage is caused by indirect mechanisms, that occurs to the cell by radiation therapy (Hall et al. 2016).

IR induces adverse oral effects such as mucositis, candidiasis, salivary gland dysfunction, xerostomia, radiation caries, osteoradionecrosis, and trismus (Tolentino et al. 2011, and Harshitha and Laliytha 2017).

IR affects bone either in high-dose radiation or in the treatment of oral cancer, and also in low-dose radiation is used in the management of long-term inflammatory or degenerative conditions, such as rheumatoid arthritis and osteoarthritis (Donaubauer et al. 2020). High-dose radiation can induce changes in the main arrangement of bone collagen, which is important for mechanical strength. It also plays a significant role in osteoblastic activity, reducing collagen production, inducing osteoblast cell cycle death, and affecting osteoblast proliferation as well as inducing a reduction of bone cells (Vissink et al. 2003, and Donaubauer et al. 2020). Osteoradionecrosis is a serious late adverse reaction of IR. It is a condition of non-vital bone that was described by Marx as the 3 ‘H’s or hypocellularity, hypovascularity, and tissue hypoxia, that occurred as a result of hyperemia, endarteritis, thrombosis, and finally the microvasculature damage (Harshitha and Laliytha 2017).

Various agents were used to reduce the hazards of IR on oral tissues; the antioxidants group is one of those agents. Antioxidants are a large group of compounds that are formed of enzymes, vitamins as ascorbic acid, α-tocopherol, and some minerals such as zinc and copper; they can prevent oxidative damage that occurs within the cell due to the release of free radicals (Hamza et al. 2018).

Ascorbic Acid, because it neutralizes the oxidative damage caused by exposure to IR by electron transfer and/or donation, vitamin C is a powerful antioxidant. (Al-Niaimi and Chiang 2017). Nowadays, vitamin C is widely used as an additive in many foods, as humans can’t create vitamin C because of the absence of L-glucono-gamma lactone oxidase enzyme (Carr and Vissers 2013, and Al-Niaimi and Chiang 2017).

Telmisartan is a highly selective angiotensin II type 1 (AT1) receptor blocker that is frequently used to treat hypertension. Telmisartan has several advantages over other AT1-receptor blockers, including increased AT1 receptor affinity, a longer half-life, and the capacity to activate peroxisome proliferator-activated receptor (PPAR), which produces an efficient antioxidant and anti-inflammatory agent. (Al-Hejjaj et al. 2011 and Goyal et al. 2011).

Furthermore, telmisartan's chemical composition includes benzoic and benzimidazolic groups, which give it special hydroxyl radical scavenging capabilities (Eslami et al. 2014). Previous researchers have documented telmisartan's protective properties against a variety of experimental models of tissue damage, demonstrating its effectiveness for clinical problems other than the treatment of hypertension. However, an ideal radioprotector should be administered in a way that is tolerable, ideally orally or intramuscularly (Cavalim Vale et al 2019, and Fooladi et al. 2022).

 This study was designed to assess the radioprotective effect of ascorbic acid versus telmisartan, besides the combination of both, on rats exposed to gamma radiation. To our knowledge, no other research has compared both agents or assessed their combination as a radioprotective agent.

## Materials and methods

### Ethical consideration

This experimental study using a rat model, the facilities, feeding, and scarification technique of the animals were all examined inv compliance with the guidelines for animal experiments authorized by the Al-Azhar University, Girls' branch ethical committee (REC-PD-24–25) and received ethical approval from the Research Ethics Committee of the National Centre for Radiation Research and Technology of The Egyptian Atomic Energy Authority in Cairo, Egypt (F/15/EC/24).

### Sample size calculation

According to study by Morales-Ramírez et al. 1998, *n* = 40 as the total sample size (35 + 10% dropout) were divided into 10 rats in each group, to determine the size of the effect Cohen f = 0.644 utilizing the G power statistical analysis tool (Version 3.1.9.4) with a real power (1-β error) of 0.8 (80%) and primary risk of error (α = 0.05) for calculating sample size and one-way analysis of variance (ANOVA).

### Animals and grouping

Forty male Albino rats weighing 200 ± 20 g and aged between 8 and 12 weeks were utilized in this investigation. Five rats per cage were kept in specially made cages with controlled environmental parameters, such as a 12-h light/dark cycle, an ambient temperature of 24–28 °C, and a relative humidity of 45–64%. Before the experiment began, the animals were fed a semi-purified food and had unlimited access to water for ten days. Rats were divided into four experimental groups at random after the acclimatization period (n = 10 per group). Fractionated gamma radiation (6 Gy) was administered to Group 1 (R) three times a week, two Gy per day. Telmisartan and fractionated gamma radiation (6 Gy) were administered to Group 2 (MR) three times a week, twice a day. Ascorbic acid and fractionated gamma radiation (6 Gy) were administered to Group 3 (CR) three times a week, two Gy per day. Telmisartan, ascorbic acid, and fractionated gamma radiation (6 Gy), 2 Gy daily, three times a week, were administered to Group 4 (MCR). Two-time intervals (three days and ten days) were used to split each group into two subgroups. This takes place at the National Center for Radiation Research and Technology, Egyptian Atomic Energy Authority, Cairo, Egypt.

#### Telmisartan application

In groups (2 & 4), 12 mg/kg of telmisartan was given orally once/day for 7 days (Micardis, Boehringer Ingelheim, Germany) (Fooladi et al. 2022).

#### Ascorbic acid application

In groups (3 & 4), 200 mg/kg of ascorbic acid was given orally once/day for 7 days (Redoxon, vitamin C 1 g, Bayer, France) (Mortazavi et al. 2015).

### Radiation exposure

With a dosage rate of 0.59 Rad/sec using 137Cs gamma cell 40, all rats were given fractionated gamma irradiation (6 Gy), 2 Gy, daily, thrice weekly (Sayed et al. 2018).

The radiation procedure was done at the Egyptian Atomic Energy Authority, National Centre for Radiation Research and Technology, Cairo, Egypt.

### Animal euthanizing

Five animals of each group were euthanized by overdose anesthesia (Ketamine 100 mg/kg) (Santos et al. 2014) at three and ten days after radiation exposure (Kondo et al. 2010) to monitor the acute phase of healing, as our study focuses on short-term intervals (3 and 10 days). Analysis of radiography, histology, and histomorphometry was used to assess the treatment's results.

### Radiographic analysis

**Fig. 1:**
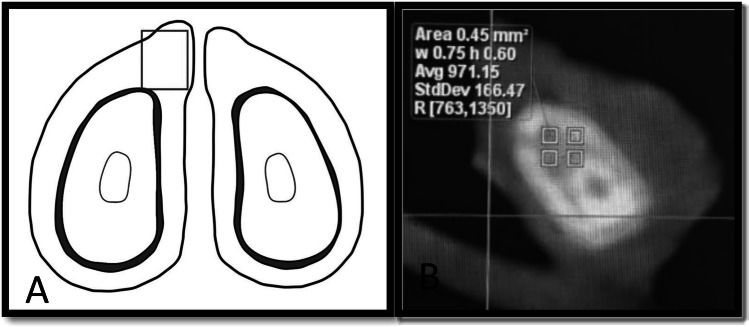
**A** A diagram by Martelli et al. 2019, showing how to measure bone density in CBCT. **B** A coronal CBCT view showing the measurement of bone density

Bone density (BD) was evaluated using (CBCT) cone-beam computed tomography. A calibrated examiner, who was blind to the group assignment, used Planmeca Romexis software to acquire and analyze the images. Each mandible was individually placed in a plastic container, with one specimen per container. Scanning parameters were standardized and recorded within the Romexis software. To ensure proper positioning and optimal scanning height, the containers were inserted into the CBCT unit with the aid of an empty positioning box. For each mandible, the anterior and posterior regions of interest (ROIs) were assessed, and a double sample identification number was assigned accordingly. The anterior ROI was defined by positioning the coronal plane at the level where bone tissue began to encircle the left incisor root, and BD measures were acquired from the bone above the root. About 2 mm away from the root to ensure that the periodontal ligament was not included. The posterior ROI was established by shifting the coronal plane distally to the final molar on the left side, where BD measurements were recorded in the bone posterior to the molar root. Two consecutive tomographic slices were analyzed for each ROI, with three measurement points evaluated per slice. The BD values from the anterior and posterior ROIs were averaged to calculate the mean BD for each mandible, following the methodology described by Martelli et al. (2019) (Fig. 1A and B).

### Histological analysis

#### Specimens’ preparation for histological and histomorphometric analysis

All mandibles were collected and, after that, harvested; preserved in 4% formaldehyde in saline buffer with phosphate for a full day, using 10% neutral buffered formalin (pH 7.4). Following fixation, they were decalcified by placing them in a 14% Ethylene Diamine Tetra-acidic Acid (EDTA) solution for 20 days; it was modified every 48 h. After that, the samples were cleaned by running water to get rid of any remaining acid (Borges et al. 2023).

#### Hematoxylin and eosin stain

The specimens were dehydrated by moving them through progressively higher alcohol concentrations before being moved to xylene to remove the alcohol. After that, the samples were inserted in the middle of blocks of paraffin wax. The embedded specimens were cut in the buccolingual plane and sectioned using a microtome to a thickness of five microns. After transferring the portions to progressively lower alcohol concentrations, distilled water was added. These slices were stained with Hematoxylin and Eosin (H & E) (Suvarna et al. 2018 and Ma et al. 2023).

#### Masson’s trichrome stain

After preparation of the slides for Hematoxylin–eosin stains, blocks were used for Masson’s trichrome stain (MT) (Yu et al. 2023).

#### Histomorphometric analysis

A computer-assisted image processing system built into a light microscope was utilized to conduct the histomorphometry analysis (Zaki et al. 2017). An oral pathologist in the Oral Pathology Department of Al Azhar University's Faculty of Dental Medicine for Girls took the resulting measurements for the regenerated bone while concealing the slides of the groups under evaluation.

**Fig. 2:**
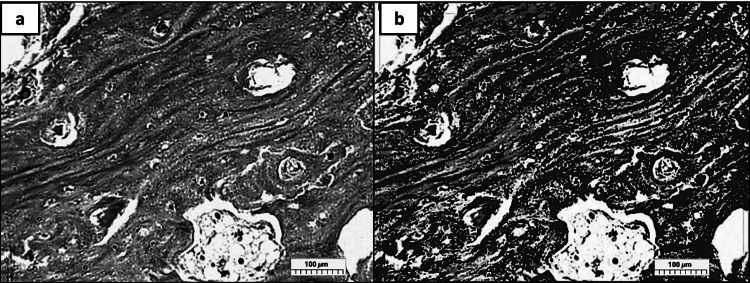
A photomicrograph showed a binary blue color that the computer could measure (**A**) before, and (**B**) after using the computer

The Leica Quin 500 analyser computer system (Leica Microsystems, England) was used to calculate the area percentage of the regenerated bone. The pointer was used to frame the bone trabeculae area, and the computer could measure the blue binary colour that was used to mark the drawn regions (Fig. 2). The image analyzer program transforms the measured units (pixels) into actual micrometre units. Next, using magnification (X 200), the area % of the regenerated bone was calculated in five distinct fields for each group. For every group, the mean and standard deviation (SD) values were computed (Delan et al. 2021).

### Statistical analysis

SPSS version 26 was used for statistical analysis, and the results were displayed as mean ± standard deviation (SD). For every statistical comparison, a significant threshold of p < 0.05 was demonstrated. The Shapiro–Wilk and Kolmogorov–Smirnov tests were used to determine whether the data distribution within each group was normal.

An independent Student's t-test was used for intra-group comparisons, and one-way ANOVA was used to evaluate inter-group differences before post hoc pairwise comparisons.

## Results

### Radiographic analysis

The bone density measurements obtained after three and ten days differed significantly for the R group (P = 0.005) and the MCR group (P = 0.01). The highest values of bone density measured after 3 days were for the MCR group (1359 HU)**,** while the lowest values of bone density measured after 3 days were for the R group (852.80 HU).

Additionally, after three and ten days, there was a substantial variation in the bone density values across the various study groups, respectively (P < 0.001) (Table 1).

**Table 1:** Represents inter- and intra-group comparisons of the bone density values after 3 days and 10 days, respectively

Group	Bone density Mean ± SD		95% confidence interval of the difference		*P* value
	After 3 days	After 10 days	Lower bound	Upper bound	
R	852.80 ± 92.02^A^	1042.60 ± 32.25^A^	−302.12-	−77.47-	0.007*
MR	1142.80 ± 40.81^B^	1121.60 ± 69.28^A^	−65.24	107.64	**0.57**
CR	1328.40 ± 136.36^C^	1233.60 ± 67.48^B^	−62.10	251.70	**0.20**
MCR	1359 ± 28.90^C^	1229.60 ± 38.35^B^	79.86	178.93	< 0.001*
*P* value	***P*** < 0.001*	***P*** < 0.001*			

*; significant (*P* ≤ 0.05), *ns* non-significant (*P* > 0.05). A statistically significant difference within the same vertical column is shown by different superscript characters

### Histological analysis

#### Hematoxylin and eosin stain results

**Fig. 3:**
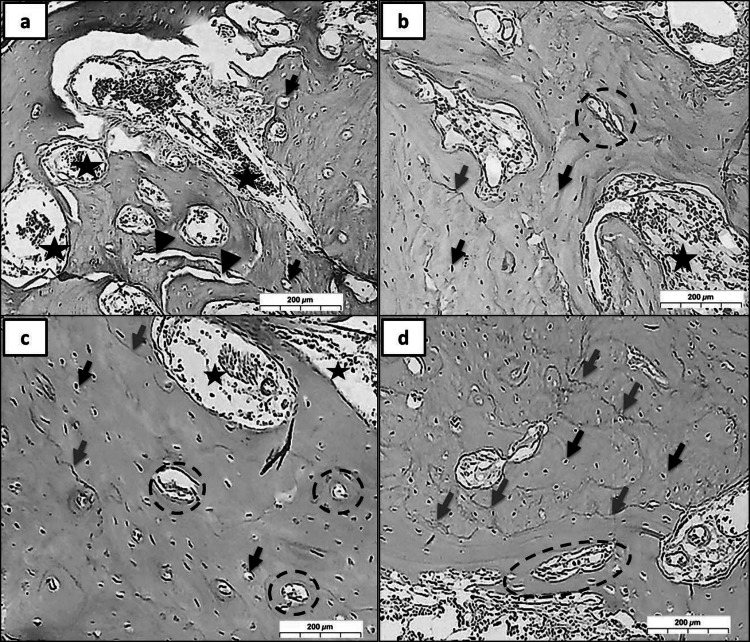
A photomicrograph showed many reversal lines and shapes of osteocytes within the lacuna *at 3 days*. (Group R; **a** with deterioration effect on the bone (arrow heads), wide osteolytic lacunae with eccentric osteocytes (black arrows), and hypervascularity (stars). (Group MR; **b** moderately normal architectures of the bone, osteocytes (black arrows), and Haversian canals (circles). (Group CR; **c** revealed a normal distribution of osteocytes with narrow lacunae. (Group MCR; **d** Many reversal lines can be easily detected (blue arrows). (H&E Orig. Mag. X 100)

In groups (R, MR, CR, and MCR at 3 Days), (Fig. 3a), as group R revealed a deterioration effect on the bone (arrow heads), some areas showed wide lacunae with eccentric nuclei (black arrows), where some regions showed normal osteocytes with normal lacunae. The haversian canal can be observed, which is surrounded by blood vessels rich with RBCs. (hypervascularity), (stars). (Fig. 3b) as group, MR showed moderately normal architectures of the bone with a relatively regular distribution of osteocytes inside the lacuna (black arrows). Many Haversian canals (circles) can be detected, which are lined with osteoblasts and filled with RBCs. While in (Fig. 3c) as group CR, the normal distribution of osteocytes with narrow lacuna, but in (Fig. 3d) as group MCR, the Haversian canals (circles) appeared wide and filled with RBCs, also many reversal lines can be easily noticed (blue arrows).

**Fig. 4:**
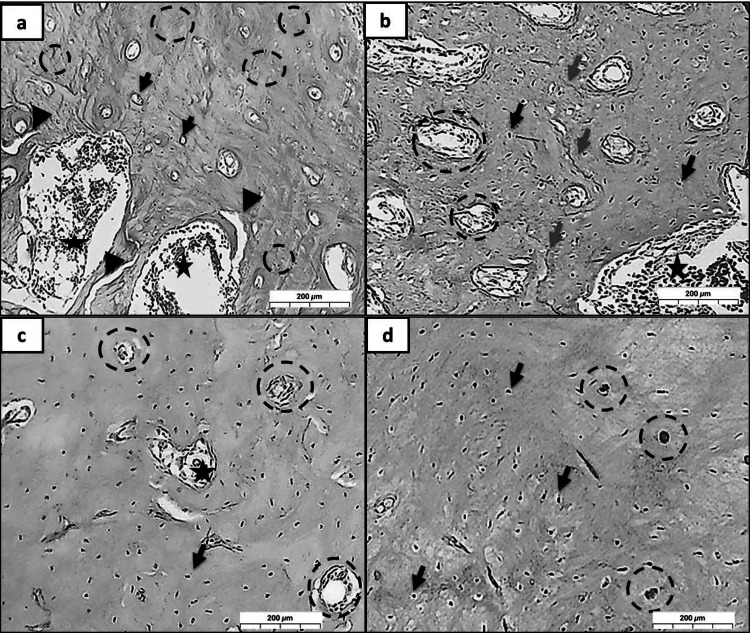
A photomicrograph showed many reversal lines and the shape of osteocytes within the lacuna *at 10 days.* (Group R; **a** showed less deterioration effect on the bone (arrow heads), empty lacunae (black arrows), absence of osteocytes (circles), and Haversian canal filled with RBCs. (stars). (Group MR; **b** showed irregular distribution of osteocytes (black arrows), (Group CR; **c** with reduced lacuna spaces (black arrows). (Group MCR; **d** displayed stenosis of Haversian canals (circles). (H&E Orig. Mag. X 100)

In groups (R, MR, CR, and MCR at 10 Days), (Fig. 4a) group R, showed a degenerating effect on the bone (arrow heads), some areas showed empty lacunae (black arrows), and other areas revealed the lack of osteocytes completely. RBCs are seen to be congested in the Haversian canal. (stars). (Fig. 4b) as group MR, showed normal architectures of the bone with irregular distribution of osteocytes, while in (Fig. 4c), as group CR, this lacuna looked constricted with reduced lacuna spaces (black arrows). However, in (Fig. 4d), there is primary evidence for true biological bone regeneration, as in group MCR, the bone is highly compact with stenosis of Haversian canals (circles).

#### Masson’s trichrome stain results

Following MT staining, the green color denotes early matrix deposition, soft collagen fibres/osteoid, whereas the red color denotes mature bone (Zhang et al. 2017).

**Fig. 5:**
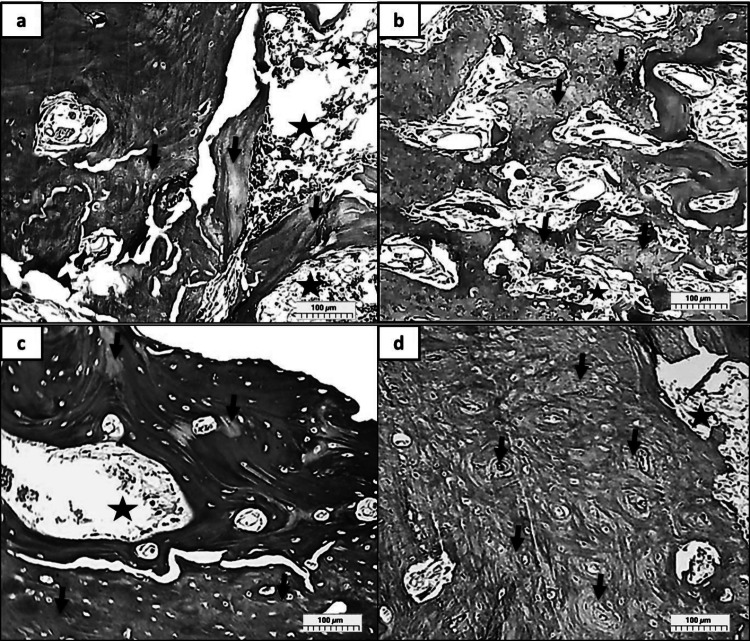
A photomicrograph showed early matrix deposition, soft collagen fibers/osteoid, not mature hard bone, in a dense green color *at 3 days***.** (Group R; **a** limited amount of early matrix deposition; (Group MR; **b** moderate amount of early matrix deposition; (Group CR; **c** large area of early matrix deposition; (Group MCR; **d** large, alternating areas of mature and early matrix deposition; regenerated bone (black arrows), inflammatory cells (stars), (MT, Orig. Mag. X 100)

In groups (R, MR, CR, and MCR at 3 Days), (Fig. 5a), the high density at 3 days likely reflects medication presence and inflammation (stars) rather than mineralized bone; in group R, limited early matrix deposition was detected with inflammatory cells; also, some fat cells were observed. (Fig. 5b), As a group, MR presented a moderate amount of early matrix deposition, and an adequate amount of fibroblastic proliferation was seen with visible osteoblastic cell proliferation. In (Fig. 5c) group CR, revealed a large area of early matrix deposition occupying many osteocytes, also some areas showed woven bone formation, especially between the mature bone, as well as fibrous marrow spaces with RBCs. However, (Fig. 5d), as group MCR, showed large, alternating areas of mature and early matrix deposition with noticeable osteoblastic cell proliferation. Many osteocytes and Haversian canals filled with RBCs can be easily detected.

**Fig. 6:**
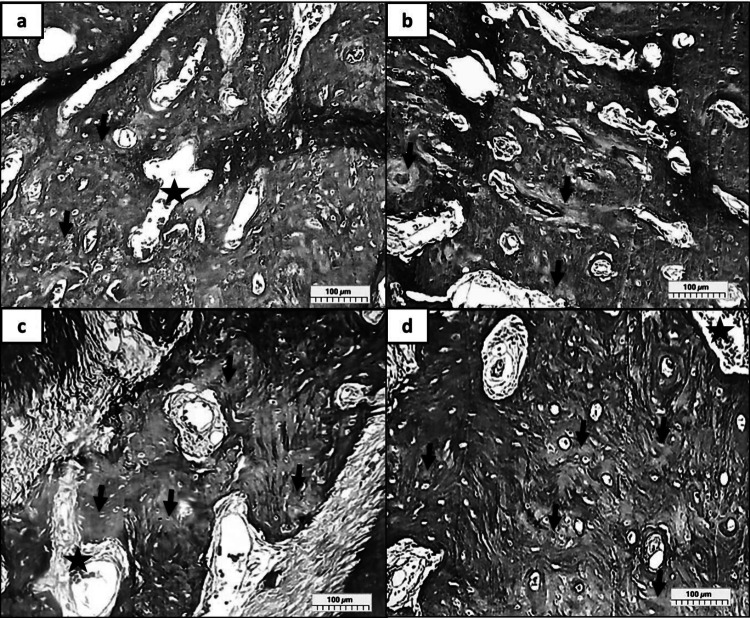
A photomicrograph showed the newly formed bone, collagen fibers, or osteoid in a dense green color *at 10 days.* (Group R; **a** small part of regenerated bone; (Group MR; **b** adequate amount of regenerated bone; (Group CR; **c** relatively large area filled with regenerated bone; (Group MCR; **e** alternating well-formed repaired bone with mature bone; regenerated bone (black arrows), inflammatory cells (stars), (MT, Orig. Mag. X 100)

In groups (R, MR, CR, and MCR at 10 Days), (Fig. 6a) group R shows a large area of mature bone enclosing a small percentage of regenerated bone. In (Fig. 6b), in group MR, the amount of regenerated bone is more compared with group R, which has a lot of Haversian canals. Whereas in (Fig. 6c), as group CR, a large area is filled with regenerated bone and collagen fibers with light inflammatory cell infiltrate. However, there is primary evidence for true biological bone regeneration, in (Fig. 6d), as group MCR, the bone is highly compact with alternating well-formed repaired bone with mature bone enclosing many osteocytes, with areas filled with RBCs as well as inflammatory cells and fibroblastic cell proliferation.

#### Area percentage of the regenerated bone

There was a significant difference between area values measured after 3 days and after 10 days for the R group (P = 0.04), the MR group (P = 0.014), and the MCR group (P = 0.001). The highest area percentage of regenerated bone was measured after 3 days for the MCR group (66.60%), while the lowest percentage was measured after 3 days for the R group (6.12%). Also, the highest area percentage of regenerated bone was measured after 10 days for the MCR group (51.12%)**,** while the lowest percentage was measured after 10 days for the R group (13.14%) (Table 2).

**Table 2:** Represents inter- and intra-group comparisons of the area percentage of regenerated bone values after 3 days and 10 days, respectively

Group	Area percentage of the regenerated bone (%) Mean ± SD		95% confidence interval of the difference		*P* value
	After 3 days	After 10 days	Lower bound	Upper bound	
R	6.12 ± 4.05^A^	13.14 ± 5.06^A^	−13.71-	−0.3322-	0.04*
MR	38.10 ± 5.09^B^	29.17 ± 3.79^B^	2.385	15.478	0.014*
CR	43.31 ± 7.00^B^	36.11 ± 12.05^B^	−7.173-	21.851	**0.281**
MCR	66.60 ± 4.23^C^	51.12 ± 4.71^C^	8.944	22.011	0.001*
*P* value	< 0.001*	< 0.001*			

*; significant (*P* ≤ 0.05); non-significant (*P* > 0.05). Within the same vertical column, a statistically significant difference is indicated by different superscript letters

## Discussion

A major interest in understanding the biology and chemistry of radiation is the identification of different radioprotective agents that can be used to prevent various oral complications resulting from head and neck radiotherapy (Zhang et al. 2023). Hence, this study aimed to assess the radioprotective benefits of ascorbic acid, telmisartan, and their combination on rats exposed to gamma radiation.

The doses of ascorbic acid (200 mg/kg) and telmisartan (12 mg/kg) were selected based on previously established radioprotective efficacy in rodent models. The ascorbic acid regimen was chosen as it represents the minimal effective dose yielding a maximal survival benefit of a life-saving effect with 90% survival in lethally irradiated rats, suggesting a potential ceiling effect (Mortazavi et al. 2015). The telmisartan dose has been validated by Fooladi et al. 2022, who reported its efficacy as a radioprotective therapy. However, dose–response relationships for either agent alone or in combination were not evaluated in the present study**.** Consequently, the observed effects are specific to the selected doses, and different dosing regimens may yield distinct biological outcomes.

The CBCT analysis for bone density of all study groups at 3 and 10 days revealed that the R group was the lowest, as radiation affects the ability of osteoblasts to produce collagen and affects osteoblastic proliferation. It was reported that IR induces the arrest of the osteoblast cell cycle (Donaubauer et al. 2020). Since a normal, biologically functioning osteoblast is essential for osteoclastic differentiation, radiation on the osteoblast will eventually indirectly affect the osteoclast, according to Mostafa et al. 2019 and El-Motasem et al. 2021. Similar results were recorded in previous studies that reported that head and neck radiation affects bone density and trabecular microarchitecture (Donaubauer et al. 2020, and Palma et al. 2020). Our study's findings also coincide with those of Sayed et al. 2018, who stated that osteoblastic resorption and a decrease in bone cells caused by gamma radiation resulted in a decrease in bone thickness. Histological analysis of the R group after 3 days revealed the deterioration effect of IR on the bone; and the ability of bone to regenerate was limited, as inflammatory cells and fat cells were detected by MT staining in this group, while after 10 days, it showed a large area of mature bone enclosing a small percentage of regenerated bone this result were in accordance with that reported by Kondo et al., 2009, that found by day 10, oxidative stressors had diminished and the bone had restored to virtually normal.

 Regarding the MR group, there were superior bone density readings in CBCT with a significant difference compared with the R group after 3 days and a non-significant difference after 10 days; this may be attributed to the radioprotective effect of telmisartan. Its anti-inflammatory impact, which inhibits prostaglandin E2, N-Methyl-D-Aspartate, and reactive oxygen species generation, as well as its radical scavenging capabilities, are responsible for its radioprotective benefits (Iin et al. 2020, and Fooladi et al. 2022). It was stated that by stimulating the internal antioxidant defence system in irradiated mice, telmisartan has been shown to enhance haematopoietic function and bone marrow repopulation (Arab et al. 2014).

Masson’s trichrome stain results for the MR group after 3 days showed the presence of a moderate amount of regenerated bone, which increased in 10 days, and an adequate amount of fibroblastic proliferation was seen with visible osteoblastic cells proliferation, and the amount of regenerated bone compared with the R group, which contained a lot of Haversian canals post-irradiation. These outcomes were consistent with those reported by Fooladi et al. (2022), who found that telmisartan improves bone marrow histology and shields bone from the deteriorating effects of radiation therapy. The tested group that received telmisartan had significantly more megakaryocytes and polymorphonuclear cells than the irradiated group.

Regarding the CR group, there were superior bone density readings in CBCT with a significant difference compared with the R and MR groups after 3 and 10 days. This is due to the radioprotective effect of vitamin C recorded in previous studies (Walder and Getoff 2014, and Mortazavi et al. 2015). They reported that ascorbic acid can reduce IR-induced damage as protein carbonylation, lipid peroxidation, cell death, lipid peroxidation, DNA double-strand breaks, and protein carbonylation.

In the CR group, after 3 days, histological analysis revealed a normal distribution of osteocytes with narrow osteolytic lacuna. Masson’s trichrome stain revealed a large area of regenerated bone occupied by many osteocytes; also, some areas showed woven bone formation, especially between the mature bone, as well as fibrous marrow spaces with RBCs. After 10 days, a large area was filled with regenerated bone and collagen fibers, with a light inflammatory cell infiltrate observed. Our results corroborate that of Sotomayor et al. 2024, who reported that ascorbic acid uses both direct and indirect antioxidant pathways to stop IR-induced DNA damage. Because of its ability to donate electrons, which enables it to oxidise to dehydroascorbate, it directly removes ROS. Additionally, it modifies the superoxide anion and nitric oxide reaction. By blocking the NF-κB pathway, ascorbic acid indirectly suppresses ROS-producing enzymes and inflammatory reactions. Furthermore, our results corroborated those of Aghajanian et al. (2015), who claimed that ascorbic acid is an essential modulator of osteoblast fate and proliferation. Genes linked to the osteoblast phenotype that can be induced following the initial deposition of collagenous extracellular matrix in osteoblast-like cells include osteopontin (OPN), osteonectin, runt-related transcription factor 2 (Runx2), alkaline phosphatase (ALP), and osteocalcin from undifferentiated mononuclear cells.

Surprisingly, the MCR group showed the highest mean bone density post-3 and 10 days, compared to all studied groups. This difference was significant for both R and MR groups, with a P value (< 0.001). This may be due to telmisartan and ascorbic acid exhibiting synergistic radioprotective effects by enhancing antioxidant, anti-inflammatory, and DNA repair mechanisms.

The histopathological analysis with Hematoxylin and eosin stain showed that the Haversian canals were wide and filled with RBCs with many reversal lines that can be easily detected post-3 days and post-10 days, showing the bone is highly compact. Masson’s trichrome stain recorded that the MCR group showed alternating areas of both mature and regenerated bone with osteoblastic cell proliferation as well as many osteocytes and haversian canals filled with RBCs with no inflammatory cells post 3 days. In contrast, the bone was highly compact with alternating well-formed repaired bone with mature bone enclosing many osteocytes, with areas filled with RBCs as well as inflammatory cells and fibroblastic cell proliferation post-10 days.

Research indicates that several cytokines, including transforming growth factor-β (TGF-β), tumor necrosis factor-alpha (TNF-α), interleukin-1 (IL-1), interleukin-6 (IL-6), and platelet-derived growth factor (PDGF), are associated with injury following irradiation. Furthermore, Angiotensin II functions as a proinflammatory cytokine, promoting inflammation by upregulating reactive oxygen species, adhesion molecules, and other inflammatory cytokines (Ekholm et al. 2009). Immunohistochemical analysis by Izu et al. 2009 demonstrated the presence of Angiotensin II type 2 (AT2) receptor protein in both osteoblasts and osteoclasts. Notably, treatment with an AT2 receptor blocker significantly increased bone mass, a phenomenon attributed to enhanced osteoblastic activity and suppressed osteoclastic activity in vivo.

Telmisartan, a modulator of the Renin–Angiotensin–Aldosterone System (RAAS), has been shown in a previous study to enhance the number of haematopoietic progenitor cells and restore bone marrow cellularity following radiation exposure (Charrier et al. 2004). The following aspects may be related to the mechanisms of action of telmisartan. First, telmisartan, a RAAS modulator, has a direct myeloprotective effect by significantly increasing the plasma content of the tetrapeptide AcSDKP (Acetyl-N-Ser-Asp-Lys-Pro-Pro). The AcSDKP is a strong myeloprotector and a regulator of haematopoiesis. It has been demonstrated that AcSDKP mediates G0/G1 cell-cycle arrest and guards against DNA damage and S-phase apoptosis (Liu et al. 2003). Telmisartan inhibit RAAS system, which is activated during radiation exposure, contributing to tissue injury as well as reducing oxidative stress by scavenging the hydroxyl radicals, inhibiting the inflammatory cytokines (Fooladi et al 2022). The second mechanism could be related to RAAS modulators' capacity to promote haematopoiesis, either directly by affecting haematopoietic stem cells during cell-cycle entrance or indirectly through increasing growth factors and cytokines released from stromal cells (Haznedaroglu and Oztürk 2003). 

The effectiveness of vitamin C as an antioxidant is partially explained by its ability to alter nuclear factor-kappa B's (NF-κB) DNA-binding activity (Cárcamo et al. 2002; Choi et al. 2004). Furthermore, vitamin C has been shown to influence angiotensin II-converting enzyme, an enzyme critical for regulating osteoclastic-osteoblastic balance(Ivanov et al. 2021). Lastly, telmisartan has been proposed to enhance hematopoietic function and marrow repopulation by stimulating the body's intrinsic antioxidant defence mechanisms within irradiated tissues, thereby counteracting the reactive oxygen species that are key contributors to radiation-induced damage(Fooladi et al. 2022).

As far as we are aware, no other histology or clinical research that are conducted to evaluate the adjunctive effect of both telmisartan and ascorbic acid combination as radioprotective agents in radiation for the head and neck, their anti-inflammatory and antioxidant qualities may be the cause of these promising outcomes (Arab et al. 2014, Eslami et al. 2014, Mortazavi et al. 2015, Cavalim Vale et al. 2019, Fooladi et al. 2022 and Gęgotek and Skrzydlewska 2022) that resulted in the synergistic effect of both agents and played a significant role on bone regeneration that were detected by histomorphometric analysis that showed that the highest area percentage of regenerated bone was recorded in the MCR group either post- 3 and 10 days.

Although the combined administration of ascorbic acid and telmisartan yielded the highest numerical values across most evaluated parameters, the difference relative to ascorbic acid monotherapy did not reach statistical significance. This observation may be attributable to a potential ceiling effect of ascorbic acid at the administered dose, given its well-established antioxidant and radioprotective properties. At this dosage, the biological response may have approached saturation, thereby limiting the measurable additional benefit of telmisartan. Nonetheless, the consistently favorable findings in the combination group support the presence of complementary mechanisms of action, which may be more clearly demonstrated with alternative dosing strategies or extended follow-up periods.

As reported before that telmisartan and ascorbic acid can act as radioprotectors in cancerous patients receiving radiotherapy, there is a debate that both can protect the tumor cells from the action of ionizing radiation. However, it was reported by Fujihara et al. 2017, Fujita et al. 2020 and Pawlowska et al. 2019, that both drugs have an anti-tumor effect that synergizes with the effect of ionizing radiation therapy, so more research is required to investigate this synergetic effect.

## Conclusions

Histological, histomorphometric, and radiographic assessments showed that when compared to telmisartan monotherapy, ascorbic acid, either by itself or in conjunction with telmisartan, produced notable radioprotective benefits. The difference between ascorbic acid alone and the combination therapy did not reach statistical significance, despite the fact that the combination regimen produced the best results. To clarify the precise function of telmisartan, both by itself and in conjunction with ascorbic acid, as possible radioprotective agents, to examine the long-term stability of the regenerated bone, and also to further study the ceiling effect of ascorbic acid, more research with larger sample numbers and longer follow-up is required. It is also advised to do additional study using varied dosage schedules for both medications.

## Data Availability

This published article contains all of the data created or examined during this investigation.

## Declarations

### Permission to publish

The Egyptian Atomic Energy Authority (EAEA) in Cairo, Egypt, approved the publication.
